# VeritaCell-Derived Autologous Skin Cell Suspensions Enhance Wound Closure Dynamics and Tissue Architecture in a Rat Excisional Wound Model

**DOI:** 10.3390/biomedicines14051079

**Published:** 2026-05-09

**Authors:** Michael Peake, Olafs Volrāts, Vladimirs Pilipenko, Jolanta Upīte, Arseniy Sergeyev, Baiba Jansone, Nikolaos T. Georgopoulos

**Affiliations:** 1 Centre for Dermatology Research, Division of Musculoskeletal and Dermatological Sciences, School of Biological Sciences, Faculty of Biology, Medicine and Health, The University of Manchester, NIHR Manchester Biomedical Research Centre, Manchester M13 9PL, UK; michael.peake@manchester.ac.uk; 2 Faculty of Medicine and Life Sciences, University of Latvia, LV-1004 Riga, Latvia; olafs.volrats@lu.lv (O.V.); vladimirs.pilipenko@gmail.com (V.P.); jolanta.upite@lu.lv (J.U.); baiba.jansone@lu.lv (B.J.); 3 VeritaCell Ltd., Kalu Street 7, LV-1058 Riga, Latvia; arseniy@veritacell.com; 4 Biomolecular Sciences Research Centre, School of Biosciences and Chemistry, Sheffield Hallam University, Sheffield S1 1WB, UK

**Keywords:** autologous cell suspensions, cell therapy, medical device, scarring, wound healing

## Abstract

**Background/Objectives**: Autologous cell suspension (ACS)-based therapy is a promising strategy to enhance wound healing, yet limitations in current methodologies hinder clinical efficacy. We have previously developed VeritaCell, a rapid isolation method that yields highly viable skin cell populations, including epidermal stem cells, and demonstrated their wound healing-enhancing biological properties in vitro (such as acceleration of keratinocyte proliferation and suppression of scarring-associated molecular responses). In the present study, we have assessed the efficacy of VeritaCell-derived ACS cell populations in enhancing both the rate and quality of healing using an in vivo rat excisional wound model. **Methods**: Full-thickness wounds were treated with ACS at donor-to-wound area ratios of 1:1, 1:10, and 1:20. Wound progression was monitored by standardised image-based quantification of percentage wound closure and healing quality was evaluated by histological assessment of tissue architecture. **Results**: ACS-treated wounds demonstrated improved early healing dynamics, with enhanced wound closure evident by Day 6 across all ACS treatment groups. Histological assessment revealed improved neo-epithelial organisation and reduced scarring-associated epidermal thickening in the 1:10 and 1:20 groups, with the 1:10 group exhibiting tissue architecture most closely resembling unwounded skin. **Conclusions**: Collectively, these findings provide preclinical validation that ACS isolates generated using the VeritaCell methodology exhibit functional activity in vivo and support improved tissue-level repair at clinically relevant donor-to-wound coverage ratios. Our observations offer insights into the strong potential of our ACS approach in providing a practical and cost-effective medical solution that will facilitate more aesthetically favourable healing outcomes.

## 1. Introduction

Breaching of the human skin barrier by insults such as burns, ulcers and physical impact causes damage which normally triggers a complex wound healing response that is driven by the spatially and temporally coordinated action of various cell types. However, wounds that are either too large (burns) or non-healing (diabetic ulcers) recover more slowly and often may be associated with microbial infection, which curtails the effectiveness of clinical wound management, thus resulting in an enormous financial burden for healthcare providers. For the National Health Service (NHS) in the UK alone, the cost of wound management is estimated to be £8.3 billion [1].

For the treatment of large wounds (such as burns), in most cases, split-thickness skin grafting (STSG) is utilised, whereby an autologous skin graft is taken from the patient and placed onto the wound site. Yet, this method can demonstrate inefficiency in healing, poor aesthetics and lengthy hospital stays [2]. Thus, there is an urgent clinical need for producing novel, alternative approaches to achieve rapid and efficient wound recovery and improvement in patient well-being. A number of alternative approaches have been developed, which include: (a) use of engineered tissues as skin ‘substitutes’ that function as barriers for prevention of fluid loss and microbial contamination [3]; (b) isolation and extensive ex vivo expansion of skin cells (keratinocytes and fibroblasts) for subsequent use in various procedures directly, such as allogeneic cell therapy [4], or by their incorporation into scaffold materials [5], or in three-dimensional bio-printed constructs for creation of physiologically functional skin structures [6]. Nevertheless, STSG still represents the current method of choice in wound care because, despite its promise, the aforementioned novel strategies have been unable to contemporaneously offer improvements in cost-effectiveness, ease of practical use, and efficacy compared to STSG.

A promising alternative strategy that can overcome such obstacles is a cell-based, “point-of-care” therapy, which involves the application of autologous skin cells. This involves enzyme-based isolation of cells from a patient’s own skin in situ, which can then be immediately applied to the wound area [7]. An advantage of such an autologous cell suspension (ACS) is the requirement of a much smaller amount of donor skin. Unlike STSG, which requires donor to wound site ratios of 1:1 or 1:2, the ACS application may require substantially less donor skin [8]. Several commercially available ACS preparation systems have been developed, including enzymatic spray-on skin approaches such as ReCell, which have demonstrated clinical utility, particularly in burn care [7,8]. However, despite such promising clinical outcomes in selected indications, broader adoption of ACS technologies has been relatively limited, not only due to procedural complexity but also due to the cost of currently available devices for ACS isolation, which remains extremely prohibitive.

To overcome the limitations of currently available methods and devices for the preparation of ACS isolates, we recently established VeritaCell for rapid, enzymatic disaggregation of human skin cells. VeritaCell provides a simplified alternative approach for rapid autologous skin-cell isolation, as it requires no specialist instrumentation and is performed at room temperature, while preserving high cell viability and recovery of regenerative cell sub-populations. We have reported efficient recovery of keratinocytes, fibroblasts and melanocytes, and VeritaCell ACS-isolates contained Epidermal Stem cells (EpSCs) and exhibited a wound healing-enhancing secretome that accelerated keratinocyte proliferation and markedly reduced cytodifferentiation of myofibroblasts, the cellular mediators of scarring [9]. Thus, our previous in vitro studies provided several lines of evidence that the established ACS isolates possess biological properties that may enhance both the speed and the ‘quality’ of wound healing.

To provide functional evidence that when such ACSs are applied to wounded skin in vivo, they can enhance neo-epithelialisation and improve the quality of the wound healing process overall, here we tested our method using the rat excisional wound model. To assess the effect of the suspensions on such acute wounds, we employed a series of donor-to-wound size ratios to unravel possible differences in efficacy, determined the rates of wound closure (neo-epithelialisation) following cell application, and examined the quality of wound recovery by assessing the organisation and uniformity of the neo-epithelium and observing for scarring-associated epidermal thickening.

## 2. Materials and Methods

### 2.1. Animals and Ethics

**Figure 1:**
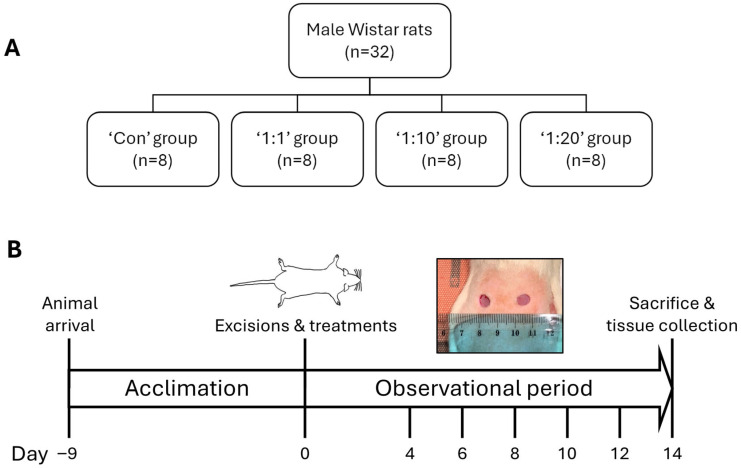
Study design. Schematic representation of the work plan to assess the effect of ACSs isolated using the VeritaCell methodology on acute wound healing in vivo. (**A**) Control and test animal groups. (**B**) Timeline of the in vivo experiments.

We recruited 32 male Wistar rats weighing 220–240 g, obtained from Charles River Laboratories (Sulzfeld, Germany). The animals were housed in environmentally enriched cages (4 rats per cage), located in individually ventilated stainless-steel racks (GR900, Tecniplast, Buguggiate, Italy). Each cage contained autoclaved aspen wood chips (1031004, LBS-Biotech, Horley, UK) in addition to enrichment items, including a polycarbonate tunnel (K3325) and aspen blocks (1023007) obtained from LBS-Biotech (UK). Rooms were maintained in accordance with experimental animal welfare regulations, under standard laboratory conditions (temperature 23 ± 1 °C; humidity 50–60%, 12 h day/night cycle 07:00 to 19:00; cage environmental enrichment) throughout the study. Rodents received a standard cereal-based pelleted chow diet (19.2% protein, 4.1% fat, 6.1% fibre and 5.9% ash) (1324, Altromin, Mucedola, Settimo Milanese, Italy) and filtered tap water supplied ad libitum. Rats were allowed to habituate to the environment of the animal facility for 9 days (Figure 1) before the experimental procedures started.

All animal experiments were conducted in accordance with applicable European regulations and national legislation governing the use of animals for scientific purposes. Experimental procedures, including study design, surgical interventions, and postoperative care, were performed in line with established ethical standards. All efforts were made to minimise animal suffering and to reduce the number of animals used.

### 2.2. Chemicals

Isoflurane was obtained from Vetpharma Animal Health (Barcelona, Spain) and buprenorphine from Le Vet Beheer (Oudewater, The Netherlands). Ethanol (70%) was supplied by Kalsnava distillery (Kalsnavas pagasts, Latvia), and Ringer’s solution was procured from Fresenius Kabi (Warsaw, Poland).

### 2.3. Experimental Design

**Figure 2:**
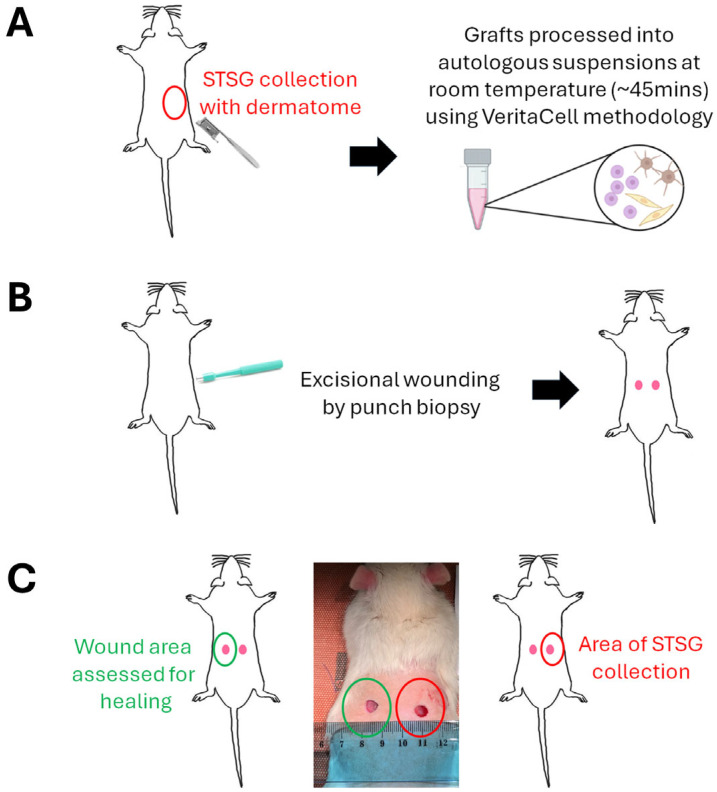
Preparation of ACS isolates for application to wounds. (**A**) STSG was excised using a dermatome from the right side of the dorsal area of each animal, and skin was processed using the VeritaCell methodology for the isolation of ACSs. (**B**) Excisional wounds were established using a punch biopsy tool. (**C**) As the punch biopsy method generates two symmetrically positioned wounds on the dorsal area, both wounds were treated with either ACS isolates or control solution. However, the donor collection area (wound indicated in red) was only utilised to perforate the skin, and it was the opposite wound (denoted in green) that was used to assess healing rates.

To assess the ability of ACS to enhance wound healing, we tested (alongside Ringer’s solution-treated ‘Controls’) three different ACS cell suspension densities (Figure 1). Prior to the establishment of excisional wounds by punch biopsy (Section 2.4), a 1–2 cm^2^ STSG was removed using a dermatome from the dorsal skin of each animal, and VeritaCell-derived ACS solutions were prepared (Figure 2). Briefly, ACS preparations were generated using the VeritaCell methodology involving optimised, rapid enzymatic disaggregation of split-thickness skin followed by controlled filtration and suspension preparation under room-temperature conditions, as fully detailed elsewhere [9].

The density of each ACS preparation was defined according to the donor-to-wound area ratio, representing the relationship between the size of the harvested donor skin and the wound area intended for treatment. Ratios of 1:1, 1:10 and 1:20 were evaluated, generated through appropriate dilution of the original ACS preparation. Thus, a 1:1 ratio indicates treatment of a wound area equivalent to the donor site size, whereas 1:10 and 1:20 represent coverage of wound areas 10-fold and 20-fold more extensive than the donor site, respectively.

For this purpose, rats were randomly divided into the following 4 treatment groups (*n* = 8 in each group): control, 1:1, 1:10 and 1:20. The treatments were applied on each wound only on day 0 (day of wound induction). After treatment administration, wounds were covered with dressing, bandage and the patch until day 4 (see below). Wound evolution was monitored by wound imaging on days 0, 4, 6, 8, 10, 12 and 14. The animals’ general health status was observed twice daily during the study period.

### 2.4. Excisional Wound Model Induction

A day before wounding, the dorsal fur of the animals was shaved with an electric clipper under anaesthesia and disinfected with 70% ethanol. On the day of wounding, rats were weighed, and to prevent acute pain, buprenorphine (0.05 mg/kg, s.c.) was injected 40 min before the surgery. Anaesthesia was induced via 4.5% isoflurane, maintained with 1.5% to 2% isoflurane in 0.3 L/min O_2_ and 0.7 L/min N_2_O, using a facemask anaesthesia system. The dorsal skin of the rat was stretched along the midline of the spine, and two circular full-thickness wounds were created with a disposable 8 mm round skin biopsy punch tool (Kai Medical, Tokyo, Japan) symmetrically along the dorsal midline of rats [10]. The formed wounds were rinsed a few times with saline. A total of two wounds were created on each rat’s back, and to avoid cross-contamination, both wounds were administered with the same treatment. Before the application of the treatment, wounds were washed with saline, and then the treatment (Control or VeritaCell-derived cell suspensions) in a volume of 100 μL was applied to both wounds (Figure 2) of each animal. Approximately 30 s after the application of the treatment, wounds were covered with one layer of a 4 cm × 4 cm piece of nonadherent, low-absorbent, small pore Telfa™ Clear Wound Dressing (Covidien, Minneapolis, MN, USA) to protect the wound area from potential infection, followed by application of an elastic self-adhesive bandage Nowopress (KOB GmbH, Wolfstein, Germany). Finally, a porous, elastic patch Mefix (Mölnlycke Health Care, Mölndal, Sweden) was wrapped around the trunk of the animal to provide a secure hold. After dressing, rats were injected subcutaneously in the back with 2 mL Ringer’s solution. After full recovery from anaesthesia, each animal was housed in an individual cage until the end of the experiment (14 days) to avoid communal licking of wounds. The dressing, bandage and patch were removed on day 4 of the study, and rats were left undressed to the open environment of their home cages.

### 2.5. Macroscopic Determination of Wound Closure Rates

As stated above, each animal received two symmetrically positioned dorsal wounds. One wound was designated as the assessment wound for imaging and closure quantification, while the contralateral wound was excluded from image analysis as the donor-site punch/perforation site for skin harvest. Therefore, wound-closure analyses were based on a single wound per animal, and the experimental (*n*) unit was the individual animal. To observe the recovery of the wound areas, wounds were photographed using a digital camera on Days 0, 4, 6, 8, 10, 12 and 14. Images were acquired using an iPhone SE (3rd generation, Cupertino, CA, USA) equipped with a 12 MP wide-angle rear camera (ƒ/1.8 aperture, 5× digital zoom, and Smart HDR 4). All images were captured under consistent lighting conditions using the same resolution (4032 × 3024 pixels) and standardised camera settings to ensure uniformity across all time points and treatment groups. Following wound image collection, the size of each wound area was measured using ImageJ software (https://imagej.net/, National Institutes of Health, Bethesda, MD, USA). The wound areas were determined by calculating the ratio of the initial area to the wound area at various time intervals. The wound margins were traced, and the % wound closure at each time point was calculated (in pixels) using the following equation and as elsewhere [11]: Wound healing rate (%) = ((Wound area on day 0 − Wound area on day (*n*))/(Wound area on day 0)) × 100%. All image analyses were carried out by a reviewer blinded to the experimental groups.

### 2.6. Histology

Tissue samples from healing wounds were collected from all animal groups under deep anaesthesia. Each tissue sample was excised with a 0.5 cm safety margin of the entire skin around the initial wound area by using scissors, with depth to the muscular fascia. The removed tissue was immediately placed in 10% (*v*/*v*) formaldehyde solution and fixed for further histological analysis. Skin samples were embedded in paraffin wax, 4 μm tissue sections prepared, dewaxed, and Hematoxylin and Eosin (H&E) staining was performed using a Dako CoverStainer automated staining system incorporating the Dako Reagent Management System (DakoRMS) (Agilent Technologies, Glostrup, Denmark). 

### 2.7. Assessment of Mean Epidermal Layer Thickness

Histochemistry microscopy images of wound tissue specimens (above) were analysed to quantify epidermal thickness across treatment and control groups using ImageJ^®^ software (https://imagej.net/). As stated above, a total of 8 animals per group were included in the study. For histological analyses, tissue sections were evaluated for technical quality prior to quantitative assessment. Sections demonstrating processing artefacts or incomplete epidermal integrity that precluded reliable measurement were excluded from such analysis. As a result, epidermal thickness quantification was performed on *n* = 7–8 animals per group, as indicated in the relevant figure captions. Within each available image, evenly spaced lines were drawn across the whole of the epidermis (to determine the distance between the basal boundary of the *stratum basale* and the apical boundary of the *stratum corneum*) within the image field of view (a minimum of 30–35 lines drawn in total per image). The lengths of these lines were measured and averaged to determine the top-down mean epidermal thickness.

### 2.8. Statistical Analysis

All experimental data are expressed as the means ± standard deviation (S.D.) or standard error of the mean (S.E.M.). Results from quantification of wound samples were averaged for each wound site; the means were then used for analysis using GraphPad Prism version 8.3.0 (GraphPad Software, San Diego, CA, USA). Comparisons between multiple groups were made using one-way or two-way ANOVA with Tukey’s post hoc test. *p*-values ≤ 0.05 were considered statistically significant (individual *p*-values within different treatment groups are appropriately indicated in the relevant figure captions).

## 3. Results

### 3.1. Study Design to Assess the Ability of ACS Isolated by the VeritaCell Methodology to Enhance Acute Wound Healing Responses In Vivo

We have previously demonstrated that our VeritaCell methodology permits isolation of highly viable human epidermal keratinocytes and fibroblasts from small STSG samples, and shown that such ACS isolates contain EpSCs that are known to support epidermal re-epithelisation and enhance the wound healing response. As the method involves enzymatic disaggregation of skin as well as filtration steps for isolation of cell populations [9], we sought to test the safety of the methodology and its ability to influence the process of wound healing in vivo.

We employed the rat excisional wound model to test the effect of our ACS on such acute wounds. As a key aim of using ACS from a small donor skin piece is to be able to ‘cover’ a several-fold larger wound area, we tested different ‘concentrations’ of such ACS isolates, to mimic different wound coverage ratios. Specifically, using an approximate ~1–2 cm^2^ donor skin size, we tested whether such isolates could benefit wounds that were 10× (‘1:10’ ratio) or 20× (‘1:20’ ratio) larger in surface area than the donor site. We tested these alongside wounds treated with the equivalent of a full cell suspension (‘1:1’ ratio) and compared ACS-treated wounds to untreated (‘Control’) wounds (Figure 1).

Of note, as the punch biopsy method generates two symmetrically positioned wounds on the dorsal area, both wounds were treated with either ACS isolate or control solution. However, as schematically represented in Figure 2 (and detailed in Section 2), the side from which donor STSG was provided to establish cell suspensions was only utilised to perforate the skin, and it was the opposite wound that was used to assess healing (detailed also in the figure caption).

### 3.2. Treatment of Acute Wounds with ACSs Enhances Wound Healing as Determined by Measurement of % Wound Closure Rates

**Figure 3:**
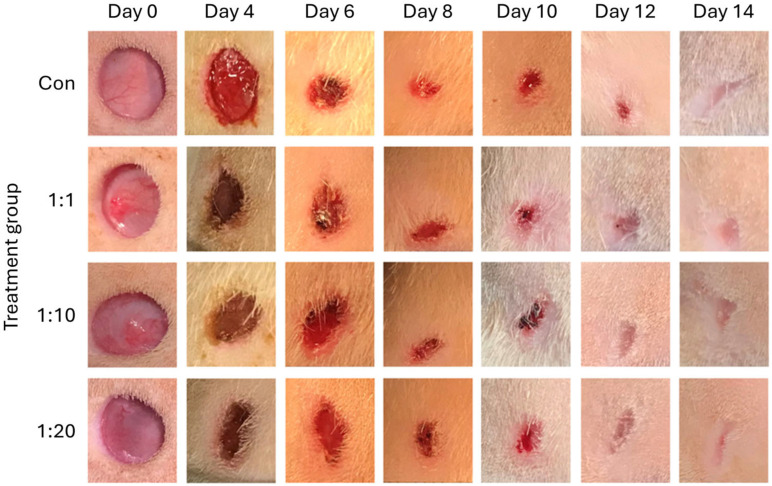
Imaging of wounds at different time points post-treatment. Excisional wounds were imaged on the way of establishment, prior to treatment with ACSs (1:1, 1:10 and 1:20) or control (Con) solution (Day 0) and application of dressings (as detailed in the Section 2). Following a period of 4 days during which wounds were undisturbed, dressings were removed (Day 4), and wounds were imaged every 2 days. Representative images of wounds throughout the 14-day assessment period are provided.

Following the establishment of punch biopsy-induced wounds and treatment with the three ACS densities (1:1, 1:10 and 1:20) or control solution (Day 0), wounds were covered with low-adherence dressings (to minimise ‘damage’ to the applied cell populations) and were left undisturbed for 4 days. Dressings were then removed, and all wound sites were imaged every 2 days thereafter until Day 14, and representative images are provided in Figure 3. Through visual inspection, it was observed that the wound tissue was in good condition in both the control and treated groups, and no signs of inflammation or wound deterioration were evident during the healing period. Yet, interestingly, we observed the clear presence of tissue exudate and a small amount of bleeding on Day 4 for Control wounds. By contrast, all wounds treated with ACS exhibited no such exudate and were visibly healthier sooner (Figure 3).

**Figure 4:**
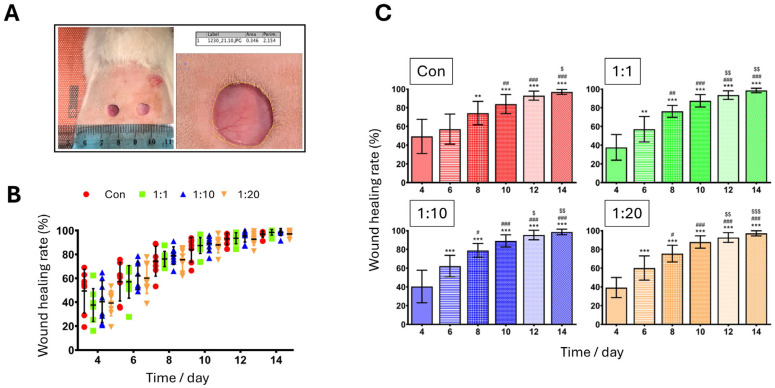
Assessment of wound closure (% healing rates). (**A**) Using ImageJ, the wound size area at each time point was determined as indicated using the software drawing tool and following ‘calibration’ of image resolution. For each treatment (Control, 1:1, 1:10 and 1:20), the measurement was repeated 3 independent times, and a mean wound ‘Area’ was calculated. (**B**) Wound closure rates (expressed as ‘% wound healing rate’) for all groups during the 14-day period were calculated (as detailed in the Section 2). Values are shown as mean ± S.D., and all individual values are presented for each group (*n* = 7–8 per group). (**C**) Wound healing rates are presented for individual treatment groups for the purposes of denoting the results of statistical analysis. Data represent mean ± S.D. (*n* = 7–8 per group). ** *p* < 0.01, *** *p* < 0.001 vs. Day 4; ^#^ *p* < 0.05, ^##^ *p* < 0.01 and ^###^ *p* < 0.001 vs. Day 6; ^$^ *p* < 0.05, ^$$^ *p* < 0.01 and ^$$$^ *p* < 0.001 vs. Day 8.

Wound area image analysis (carried out as presented in Figure 4A) permitted the determination of % wound healing rates, as shown for all groups per day of assessment in Figure 4B. Although our analysis revealed statistically significant changes between days (*p* < 0.0001), wound healing rate values did not significantly differ between groups (*p* = 0.91), indicating that for these acute, rapidly healing wounds, no significant changes in healing rates existed between groups on the days tested. However, further analysis allowed more subtle yet consistent differences to be determined when the data were assessed per treatment group.

As shown in Figure 4C, when % healing rates were analysed within each individual group, significant changes were observed and, more importantly, these patterns of significance differed between control and treatment groups. More specifically, in the Control group, a significant increase in % healing rate was observed on Days 8, 10, 12 and 14 when compared to Day 4 (*p* < 0.0001 for each); notably, however, although mean wound closure values increased numerically between Days 4 and 6 in the control group, this change did not reach statistical significance.

By contrast, in all treatment groups, a significant increase in % wound healing rate was already observed on Day 6 when compared to Day 4, as indicated by *p* < 0.01 for the 1:1 group, and even more significantly by *p* < 0.001 for both 1:10 and 1:20 treatment groups (Figure 4C), suggesting earlier progression of wound closure following ACS-based treatment. Furthermore, % healing rates in all treatment groups were significantly higher on Day 8 compared to Day 6 for groups 1:1, 1:10 and 1:20 (*p* < 0.001), in comparison to lower significance in % healing for Day 8 compared to Day 6 for the Control group (*p* < 0.01). Interestingly, particularly notable was that the 1:10 treatment group exhibited higher % wound closure at Day 6, and this group approached approximately 80% wound closure by Day 8, thus demonstrating notably strong early healing responses (Figure 4C).

### 3.3. Histological Assessment of Neo-Epithelialisation of ACS-Treated Wounds Reveals an Enhancement of the ‘Quality’ of Wound Healing and a Reduction in Scarring-Associated Epidermal Thickening

**Figure 5:**
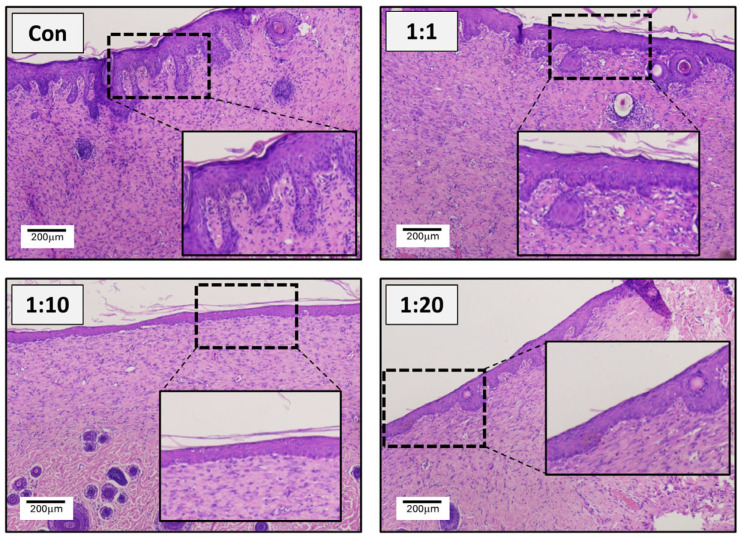
Histological examination of wounds. Following completion of the experiment time-course, on Day 14, all animals were sacrificed, and wound tissues were collected for H&E staining. Representative images from all treatments (*n* = 7–8 per group) are shown, and image inserts are included to provide a clearer representation of neo-epithelialisation. Scale bar: 200 μm.

Following completion of the wound closure experiments, tissue was collected from the wound sites and analysed by histological examination to assess the quality of the wound recovery. Inspection of the tissue architecture from representative section images shown in Figure 5 revealed striking differences between treatments. Control wounds (that were not treated with cell suspensions) showed a dense and relatively disorganised extracellular matrix in the dermis and extensive thickening of the epidermis, which was clearly highly variable in its thickness, thus indicative of a scarring-associated response. On the other hand, all wounds treated with ACS isolates exhibited better ‘quality’ of neo-epithelialisation.

**Figure 6:**
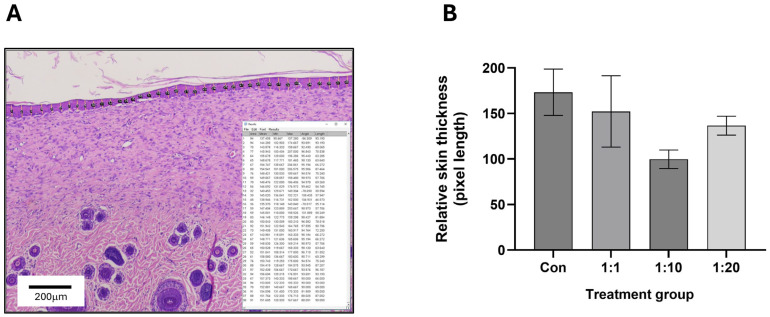
Determination of epidermal layer thickness in wounds. (**A**) Microscopy images of wound tissue histochemistry were analysed to quantify epidermal layer thickness across all treatment groups using ImageJ (as detailed in the Section 2). As indicated in the representative image, evenly spaced lines were drawn to determine the distance between the basal boundary of the stratum basale and the apical boundary of the stratum corneum across the whole of the epidermis within the field of view. The lengths of the lines were measured and averaged to determine the top-down mean epidermal thickness. (**B**) Relative skin thickness (pixel length-based) across all treatment and control groups (as represented in Figure 5) was calculated, and data are expressed as mean relative skin thickness ± SEM.

Interestingly, despite an improvement in the architecture of both the epidermal and dermal areas, 1:1 treatment was still variable in its thickness. By contrast, both the 1:10 and 1:20 treatments showed a well-organised underlying dermis and exhibited an epidermal layer that was visibly thinner and less variable in its thickness (Figure 5), with the 1:10 group demonstrating particularly reduced thickness and minimal variability (similar to unwounded intact skin). Furthermore, to provide an objective assessment of epidermal layer thickness in the treatment groups, we comprehensively assessed the epidermal layers and determined mean layer thickness (as indicated in Figure 6A). The results shown in Figure 6B confirmed that all treated wounds showed reduced neo-epithelium thickness, which was evident for the 1:20 group and was particularly striking for the 1:10 group. Although the reduction in epidermal thickness in the 1:10 group represented a clear and reproducible trend, it did not reach statistical significance (adjusted *p*-value = 0.07 using Kruskal–Wallis analysis). Of note, the 1:20 group did not demonstrate an increase in epidermal thickness relative to untreated controls; rather, the reduction in thickness was less pronounced than that observed in the 1:10 group. Overall, the pattern observed supports the interpretation that the 1:10 donor-to-wound area ratio produced the most favourable tissue architecture improvement.

## 4. Discussion

Unlike the normal process of skin re-epithelialisation by the rapid proliferation and migration of keratinocytes, deceleration of neo-epithelialisation is associated with delayed tissue repair, infection and/or chronic wound development. It is thus essential to provide solutions that isolate viable cell populations, which can then be applied directly and enhance the process of epidermal regeneration. There is increasing evidence that ACS-based therapies represent a highly promising approach [7,12], with clinical success for burns [8,13] and some promise for chronic wounds [14,15]. ACS-associated cells integrate (“take”) into a wound site [16,17], which accelerates re-epithelisation and wound healing, reduces infection risk and suppresses discomfort (pain). However, despite the promise of the ACS approach, broader clinical adoption of existing methodologies has been constrained by several practical limitations, including procedural complexity, high device-associated cost, reliance on specialised instrumentation to obtain cell populations, and uncertainty regarding optimal donor-to-wound expansion ratios [9,18,19].

To address these limitations, we have recently established VeritaCell, a novel methodology for rapid, enzymatic disaggregation of human skin cells and establishment of ACS isolates, and we examined the biological properties of these cell populations. We have reported extremely efficient recovery of human keratinocytes, fibroblasts (and melanocytes), and VeritaCell ACS-isolates contained Epidermal Stem cells (EpSCs) that were demarcated via the CD49-high/CD71-low protein expression profile. The isolated cells exhibited a wound healing-enhancing secretome which both accelerated keratinocyte proliferation and markedly curtailed cytodifferentiation of myofibroblasts, the key mediators of tissue fibrosis and scarring [9]. Thus, our previous in vitro studies implied that the established ACS isolates could support wound healing and possess biological (molecular) properties that have the potential to enhance not only the speed but, equally, the ‘quality’ of wound healing.

The primary aim of this study was to determine whether VeritaCell-derived ACS isolates exhibit measurable biological activity in an acute wound-healing model relative to spontaneous healing controls. A secondary objective was to investigate defined donor-to-wound coverage ratios as a translational framework relevant to minimising donor tissue requirements. More specifically, we sought to provide direct functional evidence that application of VeritaCell-derived ACS to wounded skin enhances neo-epithelialisation and improves the overall quality of wound repair. To address these aims, we employed a proof-of-concept rat excisional (punch biopsy-based) wound model under controlled healing conditions.

Amongst a variety of models for cutaneous wound healing assessment [20], excisional wound models in rats are widely used to study the biological processes of skin repair, despite anatomical differences between rodent and human skin. These models involve circular 8–20 mm full-thickness skin wounds (excisions) to investigate phases of healing, including inflammation, granulation tissue formation, re-epithelialization, and remodelling [21]. For wound establishment, full-thickness excisions are created on the dorsum using punch biopsy tools and wound evaluation metrics utilised include macroscopic wound area reduction tracking (via callipers or imaging) and subsequent microscopic, histological scoring of epithelialization as well as inflammation, angiogenesis, and collagen deposition [22]. Here, we created such wounds in the dorsal area and assessed the effect of VeritaCell ACS isolates at different donor-skin-size vs. wound-size ratios on wound healing. Our study employed donor-to-wound area ratios as a pragmatic translational dosing framework, reflecting the intended point-of-care clinical workflow of ACS application. Although absolute cell counts and subpopulation quantification were not determined for each preparation in this in vivo study, detailed characterisation of VeritaCell-derived ACS, including absolute skin cell numbers, cellular viability and epidermal stem cell enrichment, has been reported previously using human skin [9].

Even at the early stages of wound healing, ACS-treated wounds showed reduced exudate levels and visually demonstrated more rapid recovery. Untreated (Control) wounds exhibited gradual closure within a 14-day follow-up period, and significant healing (% wound closure) was only observed after Day 8. By contrast, treatment of wounds with ACSs of 3 different concentrations (1:1, 1:10 and 1:20) led to significant % wound closure within the first week (Days 6 and 8). Although % wound closure did not reveal striking between-group differences at matched time points, qualitative wound appearance suggested earlier improvement in skin condition in ACS-treated wounds. Macroscopic imaging captures broader healing characteristics, including exudate levels and wound-bed appearance, which may precede measurable divergence in wound area reduction. Of note, treatment with 1:10 suspensions showed particularly marked improvement in wound closure, which was the most significant throughout the healing stages. Albeit statistically significant, one may argue that these improvements may appear relatively modest. However, it must be noted that we assessed the effect of ACSs in acute and naturally rapidly healing wounds (rather than under pathological circumstances, for instance, wounds induced in a diabetic setting). Yet, even in such a biological context, by providing high numbers of viable skin cell populations, ACSs still significantly enhanced wound closure during the critical, early stages of the wound healing response. Equally, the VeritaCell process incorporates washing and filtration steps designed to minimise residual enzymatic components. No adverse inflammatory responses were observed in ACS-treated wounds, supporting the safety of the procedure.

Histological assessment of wounds provided even more notable observations. Untreated wounds on Day 14 showed a dense and visibly disorganised extracellular matrix network, and most notably exhibited extensive thickening of the neo-epidermis, which had ‘invaded’ deeply into the underlying dermis and was highly variable in its thickness, observations confirming marked epidermal thickening and indicative of a scar-associated response. Instead, upon treatment with ACS, there was improved and smoother organisation of the underlying matrix in the dermis. For both 1:10 and 1:20 treatments, the epidermal layer was thinner and far less variable in its thickness, as confirmed by detailed image analysis. Particularly striking were the observations for the 1:10 group, which demonstrated reduced thickness and such minimal variability that it was essentially histologically identical to unwounded skin. Interestingly, although one might have anticipated that the 1:1 ratio would have provided the best response (due to the provision of higher ACS cell density), clearly, the additional cell numbers did not necessarily translate to superior enhancement of the wound healing process. To our knowledge, this is the first such observations for ACS-based treatment of wounds. It is tempting to speculate that these observations may be indicative of the importance of supporting the wound to naturally resume re-epithelialisation, but without an ‘overwhelming’ (excessive) provision of autologous cells. This may also provide biological clues as to why skin grafts do not always demonstrate optimal tissue “take” and efficacy.

While the present study demonstrated clear morphological and functional improvements in wound healing following ACS treatment, it was not designed as a mechanistic dissection of dermal fibrotic signalling pathways. Comprehensive assessment of collagen deposition (e.g., Masson’s trichrome staining), myofibroblast activation (α-SMA) detection, and expression of pro-fibrotic mediators (e.g., TGF-β1) would provide additional insight into the molecular regulation of scar formation and represent important future directions. Similarly, extended follow-up (beyond 14 days employed in our study) could allow evaluation of long-term remodelling, tensile strength recovery and adnexal regeneration. Such studies would refine our understanding of the mechanisms underpinning ACS-mediated tissue repair.

Our in vivo findings confirm that treatment with VeritaCell ACS at a 1:20 donor-to-wound ratio, and even more prominently at a 1:10 ratio, not only shows acceleration of skin neo-epithelisation, but also markedly enhances the quality of wound recovery by demonstrating a reduction in scarring-associated tissue architecture. Traditionally, however, it has been suggested that although excisional wound models in rats provide valuable insights into skin repair [23], they differ from human healing processes in key anatomical and physiological aspects. Rodent wounds have been postulated to close mainly via contraction and less so by re-epithelialization, with the subcutaneous panniculus carnosus muscle driving rapid contraction, a feature absent in humans. Thus, it has often been suggested that ‘wound splinting’ is required [24] to permit assessment of scarring-associated responses. However, more recent studies have provided evidence that such excisional wounds provide a valid and reproducible wound model [21], and this permits observations on wound healing quality to be made. Attesting to the ability of such splinting-free models to provide clues on scarring-related responses is evidence generated by several in vivo rat studies demonstrating how a variety of wound response-modulating treatments influence cutaneous thickening [25,26,27,28]. Our observations on the ability of ACSs to reduce excessive, scarring-associated neo-epithelial layer thickening provide further support for the suitability of these in vivo models. Nevertheless, we acknowledge that, as splinting was not employed in the present study, the % wound closure metric may reflect a composite outcome incorporating both contraction and re-epithelialization. Notably, however, all treatment groups were equally subject to contraction forces under identical experimental conditions. Thus, relative differences in wound closure dynamics observed between groups remain valid for comparative assessment of treatment efficacy.

Furthermore, our study highlights an important advantage of the ACS methodology, which is the requirement of a much smaller amount of donor skin to enhance wound healing on a larger skin area. Unlike using STSG, which requires donor-to-wound site ratios of 1:1 (or at best 1:2), the ACS application clearly provides wound benefit by requiring remarkably less donor skin. Of note, it has previously been suggested that even a 1:80 donor site-to-wound site ratio might be clinically beneficial [8]. We would nevertheless exercise caution in ‘compromising’ the benefit of the ACS approach by ratios that are higher than 1:20. Not only are our in vivo observations supportive of a potentially ‘optimal’ donor-to-wound site ratio (and different such ACS application ratios may be utilised by clinical partitioners depending on the severity of the wounds being treated), but they may also provide an explanation as to why, so far, ACS-based therapy has not achieved the desired clinical promise (also in the context of method cost vs. clinical benefit) [18]. Notably, one key distinction of our present study is that it focused on donor-to-wound coverage optimisation using VeritaCell-derived ACS preparations. To our knowledge, our study has for the first time experimentally evaluated how donor-site expansion ratios influence tissue-level healing outcomes in vivo.

It is also important to emphasise that the therapeutic concept of ACS application in wound repair is already supported by clinical studies, including investigations using commercially available systems such as ReCell [8,13,16,17,18]. The present study was thus not designed to establish the fundamental efficacy of ACS as a promising regenerative modality per se, but rather to determine whether ACS preparations generated using the VeritaCell methodology exhibit functional activity and to explore defined, optimal donor-to-wound coverage ratios. In this context, our findings provide preclinical validation of the method. As described in our previous in vitro characterisation study [9], VeritaCell enables rapid enzymatic disaggregation of split-thickness skin at room temperature, without the use of specialist laboratory instrumentation or electrically powered incubation systems. Whilst direct comparative studies were beyond the scope of our work, the ability to achieve enhanced wound closure dynamics and improved tissue architecture using donor-to-wound ratios as low as 1:10 or 1:20 suggests that VeritaCell-derived ACS may offer practical advantages in scenarios where donor skin availability is limited, such as extensive burns.

In contrast to workflows that require device-dependent heating steps or dedicated processing platforms, our approach was intentionally designed to simplify intra-operative handling while preserving high cell viability and recovery of key regenerative sub-populations (keratinocytes, fibroblasts, melanocytes and epidermal stem cell-enriched fractions). Importantly, we previously demonstrated that these isolates exhibit biologically favourable properties, including secretion of pro-proliferative mediators and attenuation of myofibroblast cytodifferentiation [9], thus supporting not only feasibility but also functional relevance for tissue repair. The present in vivo study further extends this framework by providing biology-informed evidence to pragmatically guide donor-to-wound coverage considerations, linking defined cellular composition and high cell viability to functional healing outcomes.

## 5. Conclusions

Following on from our previous molecular findings on the beneficial biological properties of ACS-associated populations (provision of viable cell populations and a wound healing-enhancing secretome), we now demonstrate enhanced wound closure dynamics and improved tissue-level morphological characteristics, as VeritaCell suspension-treated skin showed accelerated healing rates, as well as exhibiting enhanced quality of wound healing by reducing scarring-associated epidermal tissue thickening and improving skin organisation. These preclinical observations support further investigation of VeritaCell-derived ACS as a strategy to enhance wound healing responses in critical wounds. Our findings suggest potentially strong benefits in promoting improved tissue architecture during repair and offer insights into the promise of the ACS approach in providing a medical solution that will facilitate more aesthetically favourable wound healing outcomes.

## Data Availability

The original contributions presented in this study are included in the article. Further inquiries can be directed to the corresponding author.
